# Cost-effectiveness analysis of anal cancer screening in women with cervical neoplasia in British Columbia, Canada

**DOI:** 10.1186/s12913-016-1442-2

**Published:** 2016-06-27

**Authors:** I. Cromwell, M. Gaudet, S. J. Peacock, C. Aquino-Parsons

**Affiliations:** Canadian Centre for Applied Research in Cancer Control, Vancouver, Canada; Department of Cancer Control Research, British Columbia Cancer Agency, Vancouver, Canada; Department of Radiation Oncology, British Columbia Cancer Agency, Vancouver, Canada; Division of Radiation Oncology, The Ottawa Hospital, Ottawa, Canada; Faculty of Health Sciences, Simon Fraser University, Vancouver, Canada

**Keywords:** Anal cancer, Cost-effectiveness, Screening, Cervical neoplasia

## Abstract

**Background:**

Precursors to anal squamous cell carcinoma may be detectable through screening; however, the literature suggests that population-level testing is not cost-effective. Given that high-grade cervical neoplasia (CIN) is associated with an increased risk of developing anal cancer, and in light of changing guidelines for the follow-up and management of cervical neoplasia, it is worthwhile to examine the costs and effectiveness of an anal cancer screening program delivered to women with previously-detected CIN.

**Methods:**

A model of anal cancer screening and treatment was constructed, to estimate the cost-effectiveness of a population of CIN II/III+ women who were screened using anal cytology vs. one that received no anal cancer screening. Costs were based on Canadian estimates, and survival was based on estimates taken from the scientific literature. Effectiveness was measured in terms of life years gained (LYG) and quality-adjusted life years (QALYs). The model was run for 50 cycles, with each cycle representing one year.

**Results:**

Incremental cost (screened vs. unscreened) was $82.17 per woman in the model. Incremental effectiveness was 0.004 LYG, and was equivalent to zero in terms of QALY. An ICER of $20,561/LYG was calculated, while no meaningful incremental cost-effectiveness ratio (ICER) could be calculated for quality-adjusted survival.

**Conclusion:**

Our analysis suggests that anal cancer screening is cost-effective in terms of overall survival in women with a previous diagnosis of CIN II or CIN III as part of regular follow-up, but may not contribute meaningfully-different quality-adjusted survival due to the adverse effects of screening-related interventions.

**Electronic supplementary material:**

The online version of this article (doi:10.1186/s12913-016-1442-2) contains supplementary material, which is available to authorized users.

## Background

Squamous cell carcinoma (SCC) of the anal canal is a relatively rare cancer of the gastro-intestinal (GI) tract, representing approximately 1.5 % of GI cancers [1]. Several risk factors for anal SCC (ASCC) and its precursor lesions low-grade squamous anal intraepithelial neoplasia (AIN1) and high-grade squamous anal intraepithelial neoplasia (AIN2+) have been identified including high-risk Human Papillomavirus (HPV) infection, Human Immunodeficiency Virus (HIV) infection, immunosuppression, and men who have sex with men (MSM) [2]. In women it is well established that Cervical Intraepithelial Neoplasia (CIN), often detected through cervical cancer screening, is linked to high-risk HPV subtypes and progression to invasive cervical cancer if left untreated [2–4] and multiple studies have shown that women with a past diagnosis of CIN or cervical cancer are also at increased risk of developing anal cancer [5–9]. It has also been shown that women with CIN have a much higher rate of AIN1 and AIN2+ than the general population, likely because of concurrent HPV infections of the cervical and anal mucosae [10–15].

Given that AIN1 and AIN2+ may have a lag time as long as 5–10 years (or more) [16, 17] before progressing to ASCC, early detection of pre-cancers through screening may be an effective way of reducing cancer incidence and burden. Anal cancer screening strategies have predominantly been applied to high-risk populations, mainly HIV positive MSM, but the scientific literature is not clear on the cost-effectiveness of such programs. Goldie et al. found that anal cancer screening is cost-effective in HIV positive MSM; the cost per quality-adjusted life year (QALY) is approximately $16,600 [18]. However, research by an NHS committee led by Czoski-Murray et al. [3, 19] and the Medical Advisory Secretariat from Ontario Canada [20], specifically mandated to look at cost-effectiveness of screening for ASCC, contested this finding. Both teams concluded that no subgroup, be it men or women, status HIV positive or negative, stood to benefit from anal cancer screening [3, 20]. Lazenby et al. have recently challenged this view for high-risk HIV positive women, claiming that anal cancer screening would be cost-effective in women with CD4 counts of less than 200 [21].

Despite the lack of clear consensus in the medical literature, many jurisdictions such as clinics in San Francisco and Vancouver have adopted anal cancer screening programs for HIV positive MSM and are evaluating the possibility of widening indications for screening to other risk groups such as HIV negative MSM, and women at high risk such as HIV positive women and women with a history of cervical dysplasia. These women’s risk of AIN is thought to be approximately threefold that of the normal population [11]. HIV+ women were found to have a 12 % prevalence of AIN which is similar to that of women with CIN [11]. Santoso et al. found a very similar prevalence rate of AIN at 12 % in women with CIN [13].

Because of the desire for these programs, and the changing international guidelines for the management of CIN [22, 23], this is a valuable time to examine the possibility of creating an anal cancer screening program in this population, and to explore whether or not such a program could be delivered in a cost-effective way. To our knowledge, no cost-effectiveness evaluations of an anal cancer screening program have been published concerning women with a past diagnosis of CIN, who could be considered a high-risk group.

For this reason, this study’s objective was to design a model to estimate the cost-effectiveness of adding anal cancer screening to ordinary follow-up for women with a past diagnosis of CIN.

## Methods

This population was selected because it is comprised of people who already participate in regular screening. The intervention proposed in the model is the addition of anal screening to the current follow-up schedule, rather than the creation of a new parallel screening program.

**Fig. 1:**
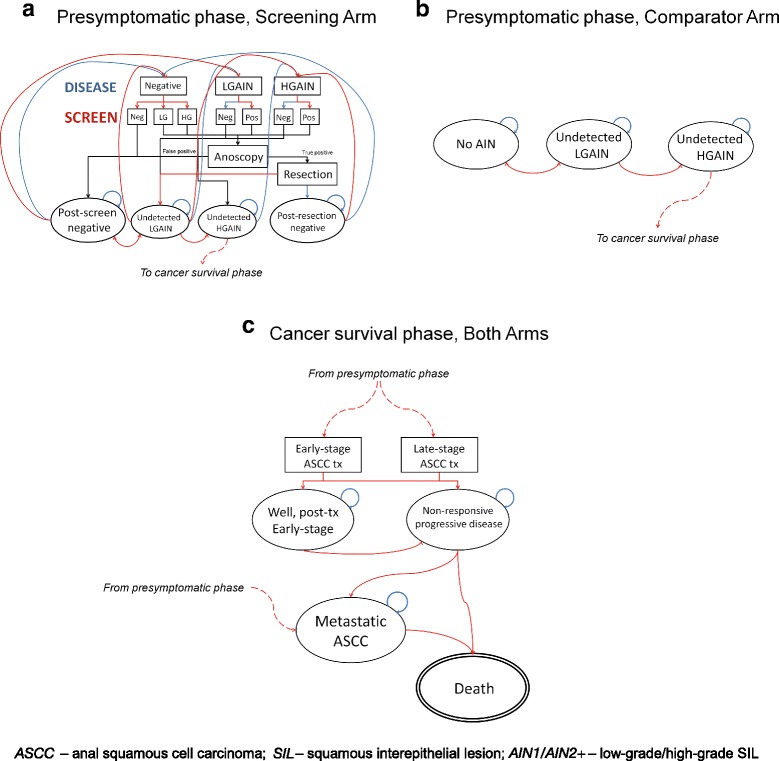
Health State Transition Model Schematic. **a** Pre-symptomatic phase, Screening Arm; **b** Pre-Symptomatic phase, Comparator Arm; **c** Cancer survival phase, Both Arms

A cost-effectiveness analysis was conducted using a Markov health state transition model of anal cancer screening and treatment. A schematic of the model is provided in Fig. 1. The model, programmed in the R environment (R Foundation for Statistical Computing, Austria), was run for 50 cycles, with each cycle representing 1 year (i.e., the model’s rull run represents 50 years of time). Each arm of the model simulated 10,000 women.

### Health state transition model

In the “Screened” arm of the model, women with a previously-detected CIN II or CIN III dysplasia are screened for AIN1 or AIN2+ via anal cytology testing. True negatives (i.e., no AIN1 or AIN2+, negative finding on screen) begin in the “Post-screen Negative” health state. False negatives (i.e., positive AIN1 or AIN2+ status, negative finding on screen) begin in the “Undetected AIN1” or “Undetected AIN2+” health states. Screen-positive women are then evaluated with high-resolution anoscopy. True positive AIN1 or AIN2+ (i.e., positive cytology status, positive finding on anoscopy) is managed via resection in the model (despite lack of consensus, the only option included in the model was resection rather than office-based resection as resection is assumed to be the more costly of the two). Women with successfully-resected dysplasia begin the model in the “Post-resection Negative” health state, and receive re-screening on the same schedule as healthy women. Women with dysplasia that is not successfully resected begin in the “Undetected AIN1” or “Undetected AIN” health states, depending on their cytology status. Women with false positive findings (i.e., negative cytology status, positive finding on screen, negative finding on anoscopy) begin the model in the “Post-screen Negative” state.

The anal screening process repeats for all women in year two and once again in year five. Follow-up screening occurs once every 3 years until year 20, after which time screening stops. The screening schedule in the model resembles screening strategies suggested by most recent international guidelines [22, 23]. This process is described in Fig. 1a.

In the “Unscreened” arm of the model, all cases of AIN1 and AIN2+ are undetected (see Fig. 1b).

Cytology-negative women (in either arm of the model) may develop AIN1. AIN1 may resolve spontaneously or progress to AIN2+. AIN2+ may progress to early-stage or late-stage anal cancer (i.e., ASCC) or present as metastatic disease. 

The model assumes that ASCC cases are managed according to British Columbia Cancer Agency (BCCA) guidelines, with both early and late-stage localized disease treated with chemoradiation therapy [24]; the disease may respond to treatment (“Well, post-Tx”) or not (“Non-responsive progressive disease”). Women with progressive disease may die within the year – those who survive may remain in the progressive disease health state, or progress to metastatic disease. Women with metastatic disease may die from cancer. The cancer survival phase of the model is presented in Fig. 1c.

### Costs

Costs to the Canadian health care system were based on estimates published in British Columbian provincial sources [24–26] and from the health economics literature when BC-specific estimates were not available [19, 27]. All cost parameters are described in Table 1. Costs were expressed in 2014 Canadian dollars, adjusted for inflation using the Consumer Price Index for health care produced by Statistics Canada [28]. Discounting was applied to all costs and outcomes at a rate of 5 % to account for future time preference, as recommended by the Canadian Agency for Drugs and Technologies in Health [29].

**Table 1:** Health State Transition Model Parameters

Parameter	Point Estimate	SE	Distribution used in PSA	Source
Transition probability				
AIN Status				
No AIN	88 %	3.2 %	Dirichlet	Santoso, 2010 [13]
AIN1	11 %	3.1 %		
AIN2+	1 %	1.0 %		
Screening utility of Pap				Santoso, 2010 [13]
Sensitivity	40 %	2.6 %	Normal	
Specificity	94 %	2.6 %	Beta	
Resection failure rate	10 %	1.0 %	Normal	Abbasakoor, 2005
AIN1 lesion recurrence rate	25 %	3.2 %	Normal	
AIN2+ lesion recurrence rate	25 %	3.1 %	Normal	
Pre-cancer survival				Palefsky, 1998 [35]
Development of new AIN1	3.5 %	1.3 %	Beta	
Remission of AIN1	3.5 %	1.5 %	Beta	
Progression from AIN1 to AIN2+	20 %	2.0 %	Normal	
Development of cancer				Adapted from Malachek 2012 [36] for HIV- MSM
AIN2+ to Early-stage ASCC	0.012 %	0.001 %	Normal	
AIN2+ to Late-stage ASCC	0.006 %	0.001 %	Normal	
AIN2+ to Metastatic ASCC	0.002 %	0.0004 %	Normal	
Cancer survival				AJCC 2010 [2, 37] Uronis 2007
Progressive disease following early-stage treatment	20 %	2.5 %	Normal	
Progressive disease following late-stage treatment	50 %	4.1 %	Normal	
Recurrence	15 %	1.2 %	Normal	
Progression to metastatic disease	50 %	2.2 %	Normal	
Death from progressive disease	80 %	4.1 %	Beta	
Death from metastatic disease	100 %	0.5 %	Beta	
Health utilities^˧^				
No AIN	0.98	0.028	Beta	Insinga, 2007 [38] ǂ
Undetected AIN1	0.98	0.024	Beta	Insinga, 2007 [38]
Undetected AIN2+	0·98	0.024	Beta	Insinga, 2007 [38]
Screen-detected lesion, resection	0·87	0.085	Beta	Insinga, 2007 [38]
Successfully-managed ASCC				
First year	0·57	0.020	Normal	Conway, 2012 [39]
Subsequent years	0·82	0.068	Beta	Melnikow, 2010 [40] ǂ
Progressive ASCC	0.57	0.020	Normal	Conway, 2012 [39]
Metastatic ASCC	0.57	0.020	Normal	Conway, 2012 [39]
Costs				
Cost of anal swab	$6	$6	Gamma	BC Cancer Agency
Cost of anoscopy	$7.55	$7.55	Gamma	BC Ministry of Health
Cost of resecting an anal lesion	$73.96	$73.96	Gamma	BC Ministry of Health
Cost of a screening appointment	$30.15	$30.15	Gamma	BC Ministry of Health
Cost of treating ASCC	$11,625	$11,625	Gamma	BCCA Ŧ
Cost of cancer follow-up appointment	$464	$46	Gamma	Tsoi, 2010 [27]
Cost of managing progressive ASCC	$18,377	$3634	Normal	Czoski-Murray, 2010 [19]
Cost of managing metastatic disease	$36,612	$7288	Normal	Tsoi, 2010 [27]

*ASCC* anal squamous cell carcinoma, *AIN* anal interepithelial neoplasia, *AIN1/AIN2+* low-grade/high-grade AIN, *CIN* cervical interepithelial neoplasia

˧ N.B. Utilities are assumed to be constant over the value of a cycle length (i.e., 1 year)

ǂ N.B. Because anal screening utility weights were not available, utilities were assumed to be similar to values found in women screened and treated for cervical lesions/cancers

Ŧ Based on cost of BCCA Chemoradiation protocols GIPART, GICART [26] – drug and administration costing data provided by BCCA Systemic Therapy Program

### Transition probabilities

Transition probabilities represent the chance that an individual will move from one health state to another (or remain in that state) from one cycle to the next. Transition probabilities are described in Table 1, along with probabilities associated with screening. These probabilities were derived from estimates in the health economic and clinical literature, and were chosen based on recency of publication, size of population studied, and applicability/appropriateness to the process being modeled.

### Quality of life (QoL)

Estimates for health state utility weights following screening and during the management of anal cancer are described in Table 1. Utilities for cervical cancer were substituted when anal cancer-specific utilities were not available.

### Cost-effectiveness analysis

The difference in total survival experienced by women in either arm of the model was calculated as Life Years Gained (LYG). Quality-weighted incremental survival was also calculated as Quality-Adjusted Life Years gained (QALYs). Incremental Cost-Effectiveness ratios (ICERs) were calculated for incremental cost per LYG and cost per QALY.

To account for the effect of uncertainty around parameter estimates, Probabilistic Sensitivity Analysis (PSA) was performed via Monte Carlo simulation [30]. Univariate Sensitivity Analysis (SA) was performed on each model parameter to investigate the extent to which incremental costs and effectiveness is changed by variations in the value of each model input. Model parameters were adjusted to 50 and 150 % of their baseline value to estimate the extent to which the ICER is sensitive to changes in each input – proportions and utilities lying close to 1.0 were adjusted by 0.1 (or less, in the case of Pap sensitivity) to reflect the underlying distribution of values.

### Estimated value of partial perfect information

The estimated value of partial perfect information (EVPPI) is a measure describing the degree to which a reduction in the uncertainty around an individual model parameter will result in a more cost-effective decision. For a given willingness to pay (WTP) for an additional year of life, it is somewhat likely that adoption of a new policy or technology (in this case, the addition of anal cancer screening to CIN II/III follow-up) will not be cost-effective due to the level of uncertainty around one or more model values. Since decisions are made on a “yes/no” basis, there is a chance that, because of parameter uncertainty, we may make a different decision than if we knew the value of the parameter with absolute certainty.

EVPPI analysis estimates the effect on the Net Benefit (incremental costs minus the value of incremental benefits, either in LYG or QALY) if we had ‘perfect’ information (i.e., zero uncertainty about the value of a given parameter), expressed as the per-person dollar value we might gain by not making a non-cost-effective decision.

EVPPI was calculated using a validated R script written by Sadatsafavi et al. [31].

## Results

**Fig. 2:**
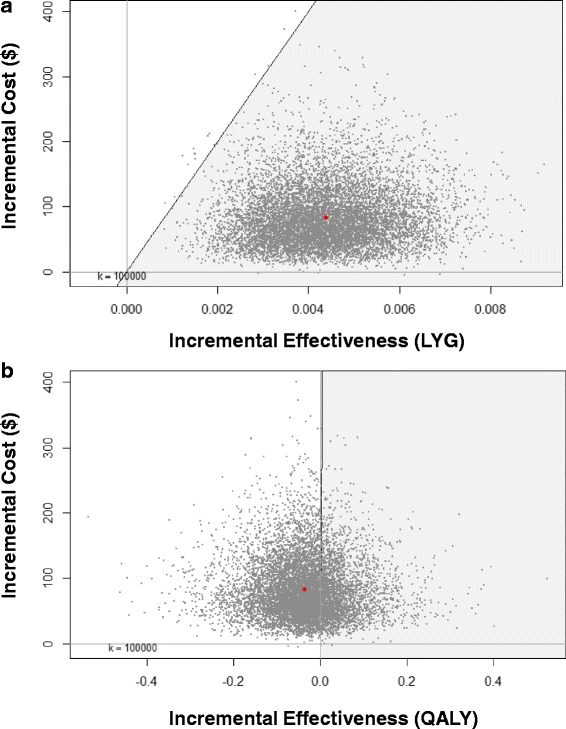
Incremental Cost-Effectiveness Plane for 10,000 Bootstrapped ICERs. **a** Cost/Life Year Gained (LYG); **b** Cost/quality-adjusted life year (QALY)

The incremental cost of the screened arm compared to the unscreened arm was $82.17 per woman. A survival difference of 4.34 × 10^−3^ (0.00434) LYG was found – this value is statistically distinct from zero. The resulting ICER was $20,562/LYG. A mean value of −0.0364 QALY was found (i.e., more quality-adjusted survival was observed in the unscreened arm), suggesting that an unscreened population has preferable quality-adjusted survival when compared to one that is screened. It must be noted, however, that this result is not statistically distinct from zero QALY (see Fig. 2).

PSA strongly suggests a great deal of uncertainty around quality-adjusted outcomes – incremental QALY estimates were greater than zero nearly as often as they were smaller than zero. The reduction in QoL associated with screening, primarily resection of low- and high-grade dysplasia, is greater than the avoided QoL loss that accompanies the reduction in cancer frequency. While the disutility for cancer is much higher, cancer is still a very rare outcome, even in this population. As a result, incremental QALYs are near (or slightly below) zero.

The change in screening policy resulted in a reduction of 7.59 cases of anal cancer per 100,000 population per year, at a cost-per-avoided-cancer of $65,403.

**Fig. 3:**
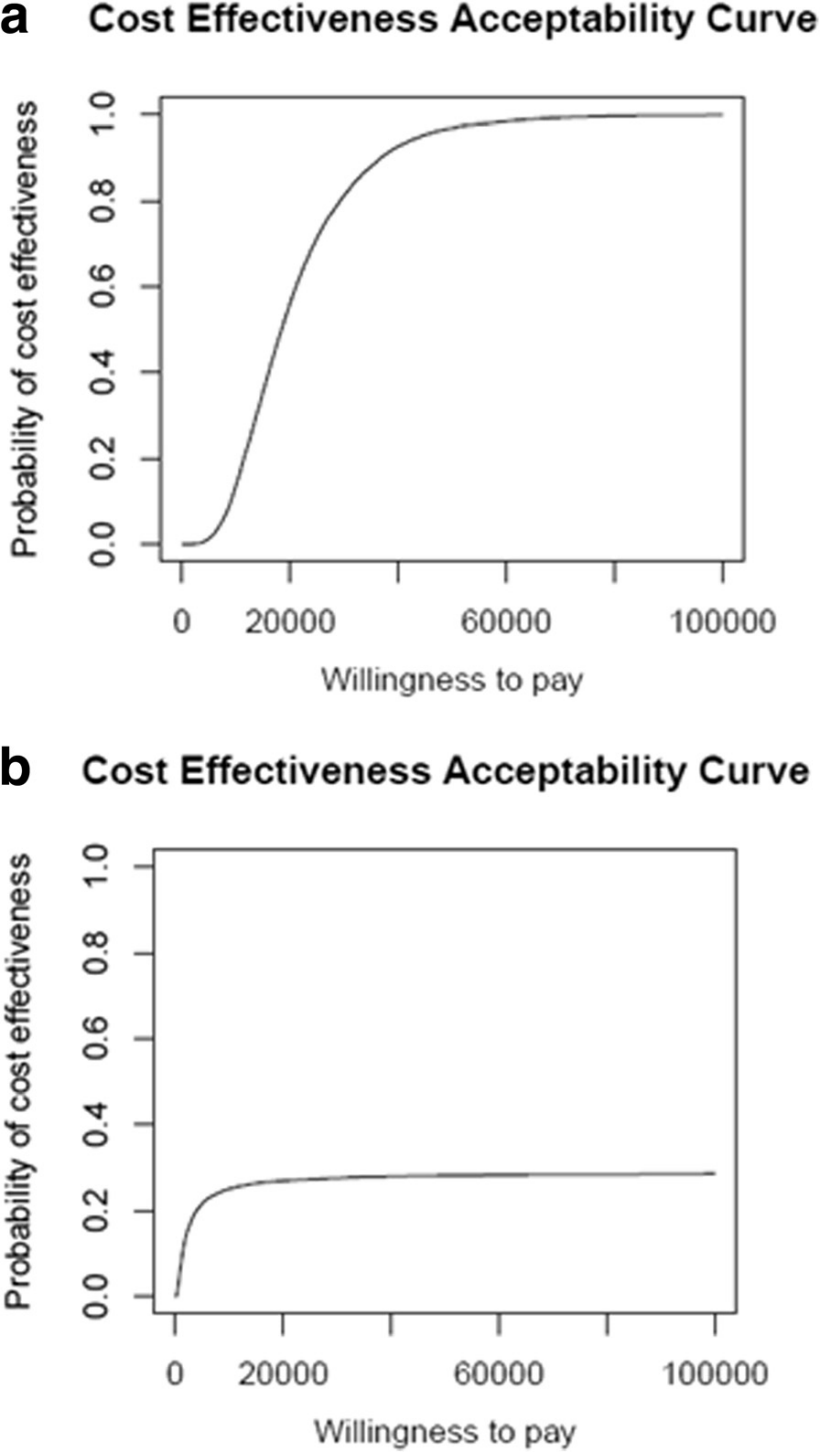
Cost-Effectiveness Acceptability Curves for Anal Cancer Screening. **a** Cost/Life Year Gained (LYG); **b** Cost/quality-adjusted life year (QALY)

Cost-effectiveness acceptability curves are shown in Fig. 3. These curves represent the proportion of model ICERs that lie below a given ‘threshold’ value that a decision-making authority would be willing to pay for a LYG or QALY. Our findings suggest that screening becomes cost-effective at a willingness-to-pay (WTP) threshold of $45,500/LYG (5 % significance level). There is no threshold at which cost/QALY reaches cost-effective at that significance level, as roughly half of probable ICERs lay to the left of the Y axis (i.e., screening was dominated).

### Alternative screening scenarios

A scenario was tested wherein women are screened for anal lesions once a year for the first 5 years after entering the model (i.e., at year zero when a CIN II/III lesion is detected – “one-off screening”). A second scenario modeled a policy where women are screened with anal cytology during their first year of follow-up only. Selected results from these alternative scenarios are presented in Table 2. The analysis suggests that, although reducing the frequency and/or intensity of screening lowers incremental cost, incremental effectiveness is also lower.

**Table 2:** Baseline and Alternative Screening Scenario Results

Scenario	ΔCost	LYG	QALY	Mean ICER	Cost/Cancer avoided
Baseline	$82.17	0.004	−0.0364	$20,561/LYG	$67,933
Scenario A – 5 years of screening	$68.25	0.002	−0.0195	$29,673/LYG; dominated^a^	$148,532
Scenario B – “One-off” screening	$13.06	0.0007	−0.0037	$52,602/LYG; dominated^a^	$102,806

LYG – incremental Life Years Gained; QALY – incremental Quality-Adjusted Life Years; ICER – Incremental Cost-Effectiveness Ratio

^a^these ICERs should be interpreted as the function of a denominator that is centered around (or slightly below) zero, rather than the result of a cost reduction

### Univariate sensitivity analysis

**Fig. 4:**
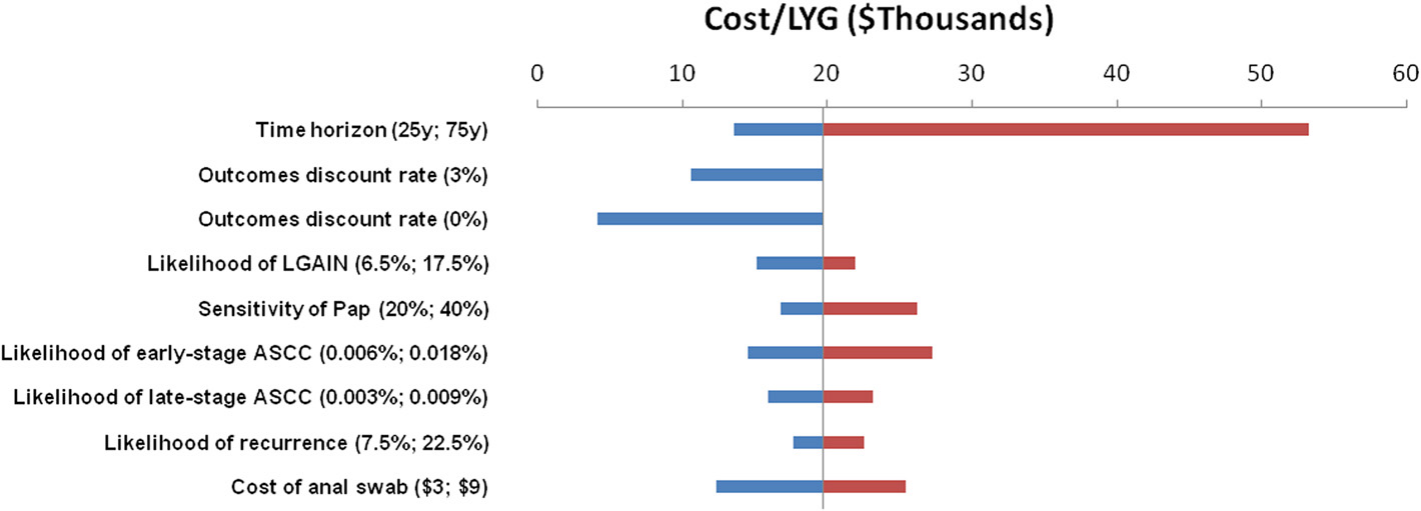
Selected Results of Univariate Sensitivity Analysis. Baseline ICER = $20,562/LYG

Selected results of this process are presented in Fig. 4. Changes in the time horizon and the discounting rate for outcomes strongly influenced the mean ICER. The model is also sensitive to the effectiveness and cost of Pap smears, as well as the incidence rate for anal cancer. A full description of the univariate sensitivity results are available in Additional file 1 attached to this manuscript.

The QALY calculation was highly sensitive to changes in utility weights for undetected AIN1 and AIN2+ (not shown) – incremental QALY adopted a positive value (i.e., was no longer dominated by a strategy with no screening) when utility weight was dropped from 0.98 to 0.90.

### Estimated value of partial perfect information

As suggested by the univariate SA, EVPPI analysis returned a threshold of zero for most model parameters (i.e., perfect information for the parameter would not result in a different decision) with a WTP threshold of $50,000/LYG. The cost of Pap testing returned an EVPPI of $0.48, and the true positive rate for screening returned an EVPPI of $0.05. This finding suggests that, although there is uncertainty around model parameter estimates, reducing this parameter uncertainty is not likely to change the decision about whether to adopt this as part of routine screening.

EVPPI was calculated for incremental quality-adjusted survival using a WTP threshold of $50,000/QALY. Based on the output of the algorithm, a value of $475.44 was found for the quality of life associated with having undetected AIN2+, suggesting that perfect information about patient quality of life may be valuable in determining whether screening is truly cost-effective in terms of quality-adjusted survival.

## Discussion

We have created a model to generate health economic evidence for whether a high-risk subpopulation – women with CIN II/III lesions – should also be screened for anal lesions. Based on the output of the model, screening CIN II/III positive women for anal cancer appears to be cost-effective in terms of overall survival, but was not different in terms of quality-adjusted survival. It is beyond the scope of this research to decide which measure – LYG or QALYs – is the ‘correct’ one to use; however, it is worth noting that the ostensible purpose of screening is to reduce the incidence of disease. It is up to policy-makers to determine which measure best reflects the priorities of their jurisdiction.

The model was sensitive to the time horizon and the outcomes discount rate. Since screening occurs only in the first 20 cycles (i.e., over the first 20 years following detecting of CIN II/III), survival and costs in both the screened and unscreened populations look increasingly similar over time. As incremental survival approaches zero, the size of the ICER increases. This suggests that the cost-effectiveness of anal screening is higher in older women than it is in younger women, but even among young women the incremental cost is still very low.

We chose to model a scenario in which anal cancer screening was conducted as part of an existing screening program, rather than the creation of an entirely new program. Given that a new screening program would be more resource-intensive than adapting an existing program, we feel that the scenario envisioned in this exercise represents the most realistic method for introducing anal cancer screening to the population. The specific method examined in this model was designed based on conversations with screening program leads in British Columbia.

Our analysis relied on the assumption that women after an anal screen experience similar quality of life to women who have undergone a cervical screen – an assumption that may not reflect the lived experience of patients. For example, cervical screening may be more normalized and therefore feel less invasive than anal screening; or perhaps anxiety about anal cancer might be greater than anxiety about cervical cancer. The accuracy of the model would likely be greatly improved by utility estimates for people following screening for anal cancer; however, these estimates are not readily available from the scientific literature. Univariate SA and EVPPI analyses suggest that the estimate of quality-adjusted cost-effectiveness would be improved by more precise information about health utility of people with untreated AIN2+.

The model also assumes that a woman who has had a resection for an anal lesion has a health disutility (loss of utility) that is constant over the course of a year; however, it is perhaps more likely that after the initial discomfort associated with anoscopy and excision dissipates, women return to a normal state of health earlier than 12 months. If this is the case, screened women would likely have *less* QoL reduction than our model estimates, resulting in a more favourable incremental QALY estimate. By allowing the QoL value to vary, our model accounts for the effect of this uncertainty, albeit indirectly.

Our model suggests that while screening this population for anal cancer may not lead to a large reduction in mortality at the population level, the additional cost is very low compared to other screening interventions that see wider utilization [32].

Women with CIN II/III lesions are more likely to develop cervical cancers than a sample drawn from the general population. This risk has been estimated to be as high as 1.6 % over 10 years and 12 % at 20 years [33], with a risk of cervical cancer that is approximately sixfold that of the general population [4]. The incidence rate of invasive cervical cancer in women with CIN was estimated to be 37 cases per 100,000 person-years in BC women with CIN. This risk is substantially higher than their risk of anal cancer which in BC women was estimated to be approximately 3.6 cases per 100,000 person-years (vs. an expected rate of 2 in the general population) [34], but the risk of progression of AIN2+ to anal cancer is thought to be 9–13 % over 10 years [16]. Seeing as their risk of cervical cancer is much greater than that of anal cancer, our model attempted to mirror their continued screening for cervical cancer rather than add to it. Our model does not include death by cervical cancer as a possible outcome, given that the risk of cervical cancer is equal between the screened and unscreened arms. This means that, although the model will likely overestimate *absolute* costs and survival for a ‘real-world’ population of screened women, the *incremental* costs and survival are not likely to change (since an equal number of cervical cancers can be expected in both screened and unscreened women).

Our model also does not account for recurring CIN II/III – it is possible that a woman’s cervical lesions return following the original detection in year 0, which would change the follow-up screening interval. A screening strategy that includes the probability of CIN recurrence would mean that the time horizon of screening would be longer than 20 years, and the cost (and cancers avoided) would increase as a result. We chose not to model this event, as we felt it would greatly increase the complexity of the model without adding much useful information – a fairly small proportion of screened women with have a recurrent CIN II/III lesion, and will therefore contribute a comparatively small difference in costs and effectiveness that is unlikely to affect the overall cost-effectiveness of adoption. We feel that any difference due to recurrent CIN II/III is likely accounted for in the sensitivity analysis, and would likely not change the decision implication of this study (i.e., screening would not likely become non-cost-effective if CIN II/III recurrence was included in the model).

For similar reasons of feasibility, we did not include women with a history of cervical cancer, or who had cervical cancer at the time of diagnosis. Treatment of comorbid cancers (i.e., cervical and anal cancer) is far more complex than our model can simulate. Women with comorbid cancers will represent a very small percentage of our target population, and their inclusion is unlikely to strongly affect our estimates of cost-effectiveness.

Our model was limited by the availability of data to inform transition probabilities and utilities. In order to allow comparability between our exercise and the existing literature, we used on parameters and values from previously-published studies. Because of the lack of health utility estimates for anal cancer screening, we assumed that utility values for AIN are similar to those for CIN. Our sensitivity analysis suggests that further research, particularly with regard to post-resection utility, may lead to a change in the decision recommendation made from this model.

We believe our model is novel in that it incorporates an anal cancer screening strategy similar to most recent published guidelines for cervical cancer screening and follow-up of cervical dysplasia. Most other analyses of anal cancer screening had attempted to model annual screening, which in most cases was shown not to be cost-effective [18, 19–21]. Lazenby et al. utilized a biennial screening strategy that was shown to be cost-effective in women with advanced HIV [21]. Our model, which uses natural history inputs and structural assumptions derived from these previously-published studies, proposes capitalizing on existing screening/followup infrastructure, rather than creating a novel program. Even given the differences between the modeled populations (otherwise-healthy women with CIN II/III) and the treatment regimes (reflecting the British Columbia health care system) between our model and those published in the cited literature, we feel that the results from this exercise support a recommendation that an anal cancer screening strategy can be adapted to a population already being screened. Given that our analysis suggests that women may experience disutility due to screening, the decision to implement a screening program ought to consider other factors, such as stakeholder preference and the health care system’s overall cancer control strategy.

## Conclusions

The addition of anal Pap cytology to regular follow-up for women with detected CIN II/III lesions was shown to be 95 % cost-effective at a WTP threshold of $45,500/LYG. The low incremental effectiveness in terms of LYG is matched by low incremental costs. The analysis did not find a difference in terms of quality-adjusted survival, suggesting that it may not be cost-effective when QALYs are considered.

This study demonstrates that anal cancer screening, while not likely to be cost-effective at the level of the general population, may be useful in populations with a higher risk of anal cancer who are already regular users of health care resources. Health care systems should consider anal screening as a low-cost method of cancer prevention in women with previously-detected CIN II or CIN III.

## Abbreviations

SCC, squamous cell carcinoma; GI, gastro-intestinal; ASCC, anal squamous cell carcinoma; AIN1, low-grade anal intraepithelial neoplasia; AIN2+, high-grade anal intraepithelial neoplasia; HPV, human papillomavirus; HIV, Human Immunodeficiency Virus; MSM, men who have sex with men; CIN, cervical intraepithelial neoplasia; QALY, quality-adjusted life year; QoL, quality of life; LYG, life years gained; ICER, incremental cost-effectiveness ratio; PSA, probabilistic sensitivity analysis; SA, sensitivity analysis; WTP, willingness to pay; EVPPI, expected value of partial perfect information; EVPI, expected value of perfect information.

## Additional file

Additional file 1: Full Results of Univariate Sensitivity Analysis and Expected Value of Partial Perfect Information (EVPPI). (DOC 110 kb)
