# Supplementary material for: Cost-effectiveness analysis of anal cancer screening in women with cervical neoplasia in British Columbia, Canada
Source: BMC Health Serv Res. 2016 Jun 27;16:206. doi: 10.1186/s12913-016-1442-2 (PMC4924299; doi:10.1186/s12913-016-1442-2)
Supplement: Additional file 1: — Full Results of Univariate Sensitivity Analysis and Expected Value of Partial Perfect Information (EVPPI). (DOC 110 kb) [file 12913_2016_1442_MOESM1_ESM.doc]

**Table S1: Univariate Sensitivity Analysis Results**

This table describes the values informing the model, and the results of univariate sensitivity analysis.

| **Model Parameter** | **Baseline Value** | **Lower Bound** | **Value (% change)** | **Upper Bound** | **Value (% change)** |
| --- | --- | --- | --- | --- | --- |
| *Probabilities* | *Reference value: $20,026/LYG* | | | | |
| AIN Status |  |  |  |  |  |
| No AIN | **0.88** | 0.44 | **17,857 (-12.2)** | **0.98** | **22,695 (+13.3%)** |
| AIN1 | **0.11** | 0.065 | **21,954 (+9.6)** | **0.175** | **18,387 (-9.0)** |
| AIN2+ | **0.01** | 0.005 | **20,351 (+1.6)** | **0.015** | **19,386 (-3.3)** |
| Pap Specificity | **0.94** | 0.47 | **19,959 (-0.04)** | **0.98** | **20,347 (+1.6)** |
| Pap Sensitivity (AIN1) | **0.4** | 0.2 | **31,841 (+58.9)** | **0.6** | **17,595 (-13.9)** |
| Pap Sensitivity (AIN2+) | **0.4** | 0.2 | **19,634 (-2.0)** | **0.6** | **20,154 (+0.6)** |
| Resection Failure Rate | **0.1** | 0.05 | **19,030 (-5.3)** | **0.15** | **21,481 (+7.2)** |
| AIN1 Recurrence Rate | **0.25** | 0.125 | **17,131 (-16.9)** | **0.375** | **23,059 (+15.1)** |
| AIN2+ Recurrence Rate | **0.25** | 0.125 | **15,455 (-29.6)** | **0.375** | **29,374 (+46.6)** |
| Progression from AIN1 to AIN2+ | **0.2** | 0.1 | **30,782 (+53.7)** | **0.3** | **17,493 (-14.5)** |
| Development of new AIN1 | **0.035** | 0.0175 | **19,354 (-3.5)** | **0.0525** | **20,968 (+4.7)** |
| Remission of AIN1 | **0.035** | 0.0175 | **19,751 (-1.4)** | **0.0525** | **20,037 (+0.002)** |
| AIN2+ to early-stage ASCC | **0.00012** | 0.00006 | **28,482 (+42.2)** | **0.00018** | **14,501 (-38.1)** |
| AIN2+ to late-stage ASCC | **0.00006** | 0.00003 | **23,758 (+18.6)** | **0.00009** | **18,785 (-6.6)** |
| AIN2+ to Metastatic ASCC | **0.00002** | 0.00001 | **22,147 (+10.5)** | **0.00003** | **18,028 (-11.1)** |
| Progressive disease after early-stage treatment | **0.2** | 0.1 | **21,119 (+5.4)** | **0.3** | **18,788 (+6.6)** |
| Progressive disease after late-stage treatment | **0.5** | 0.25 | **20,493 (+2.3)** | **0.75** | **19,608 (-2.2)** |
| Recurrence | **0.15** | 0.075 | **26,503 (+32.3)** | **0.225** | **18,379 (-9.0)** |
| Death from progressive disease | **0.8** | 0.4 | **19,997 (-0.2)** | **1.0** | **20,341 (+1.5)** |
| Progression to metastatic disease | **0.5** | 0.25 | **20,205 (+0.9)** | **0.75** | **19,460 (-2.9)** |
| Death from metastatic disease | **1** | 0.5 | **19,716 (-1.6)** | **N/A** | **N/A** |
| *Utilities* | *Reference value: -0.0385 QALY* | | | | |
| First year after negative screen | **0.9** | 0.8 | **-0.0361 (+6.6)** | **1.0** | **-0.0383 (+0.5)** |
| Negative screening result | **0.98** | 0.97 | **-0.0584 (-52.8)** | **0.99** | **-0.01759 (+118.8)** |
| Undetected AIN1 | **0.98** | 0.97 | **-0.0368 (+4.6)** | **0.99** | **-0.0385 (-0.05)** |
| Undetected AIN2+ | **0.98** | 0.96 | **-0.0138 (+178.8)** | **0.99** | **-0.0624 (-62.2)** |
| Resection following detected lesion | **0.87** | 0.82 | **-0.0576 (-49.7)** | **0.93** | **-0.0127 (+203)** |
| First year following ASCC treatment | **0.57** | 0.29 | **-0.0379 (+1.5)** | **0.86** | **-0.0351 (+9.6)** |
| Subsequent years after ASCC treatment | **0.82** | 0.73 | **-0.0412 (-7.1)** | **0.91** | **-0.0384 (+0.2)** |
| Progressive ASCC | **0.57** | 0.29 | **-0.0375 (-2.6)** | **0.86** | **-0.0337 (+14.2)** |
| Metastatic ASCC | **0.57** | 0.29 | **-0.0393 (-2.1)** | **0.86** | **-0.398 (-3.4)** |
| *Costs* | *Reference value: $82.80* | | | | |
| Anal swab | **6** | 3 | **69.16 (-19.7)** | **9** | **150.2 (+81.1)** |
| Anoscopy | **7.55** | 3.78 | **66.76 (-24.0)** | **11.33** | **87.57 (+5.8)** |
| Resecting anal lesion | **73.96** | 36.98 | **66.57 (-24.4)** | **110.94** | **98.87 (+19.4)** |
| Screening appointment | **30.15** | 15.08 | **83.29 (+0.5)** | **45.23** | **83.27 (+0.6)** |
| Treating ASCC | **11625** | 5812.50 | **82.23 (-0.7)** | **17437.5** | **82.3 (-0.6)** |
| Follow-up appointment | **464.14** | 232.07 | **81.84 (-1.2)** | **696.21** | **83.29 (+0.6)** |
| Management of progressive ASCC | **18376.27** | 9188.14 | **85.41 (+3.2)** | **27,564.41** | **78.41 (-5.6)** |
| Management of metastatic ASCC | **36611.64** | 18305.82 | **83.49 (+0.8)** | **54,917.46** | **79.07 (-4.7)** |

Note: reference values differ slightly from values presented in the manuscript due to the probabilistic nature of the parameter estimates.

**Table S2: Results of EVPPI**

| **Model Parameter** | **EVPPI ($)** |
| --- | --- |
| Probabilities | |
| No AIN | 0.05 |
| AIN 1 | 0 |
| AIN 2+ | 0 |
| Pap specificity | 0 |
| Pap sensitivity (AIN1) | 0 |
| Pap sensitivity (AIN2+) | 0 |
| Resection Failure Rate | 0 |
| AIN1 Recurrence Rate | 0 |
| AIN2+ Recurrence Rate | 0 |
| Progression from AIN1 to AIN2+ | 0 |
| Development of new AIN1 | 0 |
| Remission of AIN1 | 0 |
| AIN2+ to early-stage ASCC | 0 |
| AIN2+ to late-stage ASCC | 0 |
| AIN2+ to Metastatic ASCC | 0 |
| Progressive disease after early-stage treatment | 0 |
| Progressive disease after late-stage treatment | 0 |
| Recurrence | 0 |
| Death from progressive disease | 0 |
| Progression to metastatic disease | 0 |
| Death from metastatic disease | 0 |
| Health State Utilities | |
| First year after negative screen | 0 |
| Negative screening result | 0 |
| Undetected AIN1 | 0 |
| Undetected AIN2+ | 475.44 |
| Resection following detected lesion | 0 |
| First year following ASCC treatment | 0 |
| Subsequent years after ASCC treatment | 0 |
| Progressive ASCC | 0 |
| Metastatic ASCC | 0 |
| Costs | |
| Anal swab | 0.48 |
| Anoscopy | 0 |
| Resecting anal lesion | 0 |
| Screening appointment | 0 |
| Treating ASCC | 0 |
| Follow-up appointment | 0 |
| Management of progressive ASCC | 0 |
| Management of metastatic ASCC | 0 |
